# Spectrum of Cardiac Complications in Diabetic Patients in Rural Uttar Pradesh, India: A Hospital-Based Study

**DOI:** 10.7759/cureus.99734

**Published:** 2025-12-20

**Authors:** Manoj Kumar, Vijay K Verma, Subhash Chandra

**Affiliations:** 1 General Medicine, Uttar Pradesh University of Medical Sciences, Saifai, IND; 2 Cardiology, Uttar Pradesh University of Medical Sciences, Saifai, IND

**Keywords:** cardiac complications, coronary artery disease, diabetes mellitus, diabetic cardiomyopathy, diastolic dysfunction

## Abstract

Background: Diabetes mellitus significantly contributes to cardiovascular morbidity, particularly in rural populations with limited healthcare access. This study investigated the spectrum of cardiac complications among individuals with diabetes in rural Uttar Pradesh, India, along with associated risk factors.

Methodology: A hospital-based study was conducted at the Department of General Medicine, Uttar Pradesh University of Medical Sciences (UPUMS), Saifai, Etawah, from March 2024 to March 2025. A total of 150 diabetic patients attending the outpatient department (OPD) were assessed for cardiac complications using clinical findings, baseline electrocardiogram, treadmill stress test, and four-dimensional (4D) echocardiography. Glycosylated hemoglobin (HbA1c) was measured via high-performance liquid chromatography. Statistical analyses included one-way analysis of variance with Tukey's honestly significant difference (HSD) post hoc tests for HbA1c and duration of diabetes, as well as multinomial logistic regression to evaluate risk factors.

Results: Of 150 patients, 99 (66%) had cardiac complications. Among those with cardiac complications, 34 (34%) had coronary artery disease (CAD), 33 (33%) had diastolic dysfunction, and 32 (32%) had diabetic cardiomyopathy. Analysis of variance showed significant differences in HbA1c (F=3.70, p=0.013) and duration of diabetes (F=16.73, p<0.000001) across groups. Post hoc tests indicated CAD patients had significantly higher HbA1c and longer diabetes duration. Multinomial logistic regression identified higher HbA1c, low-density lipoprotein cholesterol, triglycerides, total cholesterol, age, duration of diabetes, hypertension, smoking, and sedentary lifestyle as significant CAD risk factors (p<0.05).

Conclusions: CAD was associated with elevated HbA1c, prolonged diabetes duration, and multiple modifiable risk factors. Early screening and targeted interventions are essential for managing cardiac complications in rural diabetic populations.

## Introduction

Diabetes mellitus represents one of the most pressing global health challenges of the 21^st^ century, often described as a silent epidemic due to its insidious onset and far-reaching complications. According to the International Diabetes Federation, approximately 537 million adults worldwide were living with diabetes in 2021, a figure projected to rise to 783 million by 2045 [1]. India, often dubbed the diabetes capital of the world, bears a disproportionate burden, with an estimated 77 million individuals affected, making it the second-highest globally after China [2]. This prevalence is particularly alarming in rural areas, where socioeconomic disparities, limited healthcare infrastructure, and cultural barriers exacerbate the disease's impact.

In the context of Uttar Pradesh, India's most populous state with over 240 million residents, rural populations constitute about 77% of the total, yet they have the least access to specialized medical care [3]. Rural Uttar Pradesh, characterized by agrarian economies, low literacy rates (around 67% for adults) [4], and high poverty levels, presents unique hurdles in diabetes management. Delayed diagnosis is commonplace, with many patients presenting only when complications manifest, often after years of uncontrolled hyperglycemia. Studies indicate that rural Indians are diagnosed with diabetes nearly a decade later than their urban counterparts, leading to accelerated progression of comorbidities [3].

Among the myriad complications of diabetes, cardiovascular diseases stand out as the primary cause of mortality, accounting for up to 65% of diabetes-related deaths [5]. The triad of coronary artery disease (CAD), diastolic dysfunction, and diabetic cardiomyopathy forms the spectrum of cardiac complications that plague people with diabetes. CAD, characterized by atherosclerotic plaque buildup in coronary arteries, leads to myocardial ischemia and infarction. Diastolic dysfunction involves impaired ventricular relaxation, often an early harbinger of heart failure with preserved ejection fraction. Diabetic cardiomyopathy, a direct myocardial injury from chronic hyperglycemia, results in fibrosis, hypertrophy, and systolic dysfunction independent of CAD or hypertension [6].

The pathophysiology linking diabetes to these cardiac issues is multifaceted. Chronic hyperglycemia induces advanced glycation end-products, oxidative stress, and inflammation, which damage endothelial cells and promote atherosclerosis [6]. Dyslipidemia, a hallmark of type 2 diabetes, features elevated triglycerides, low high-density lipoprotein cholesterol, and small, dense low-density lipoprotein particles that are particularly atherogenic. Hypertension, prevalent in over 50% of diabetic patients, further strains the myocardium through increased afterload and concentric hypertrophy [7]. Lifestyle factors such as smoking, sedentary behavior, and poor dietary habits, common in rural settings due to agricultural labor patterns and limited recreational facilities, compound these risks [8].

In rural Uttar Pradesh, these factors are amplified by systemic issues. Healthcare access is sparse, primary health centers are understaffed, and specialist consultations require arduous travel to district hospitals. A study by the Indian Council of Medical Research-India Diabetes highlighted that rural India has a diabetes prevalence of 5.6%, yet awareness and treatment rates lag at 24% and 17%, respectively [2]. Microvascular complications like retinopathy, neuropathy, and nephropathy often coexist with macrovascular cardiac issues, forming a vicious cycle that diminishes quality of life and increases economic burden on families already strained by out-of-pocket expenses [9].

This hospital-based study, conducted at Uttar Pradesh University of Medical Sciences (UPUMS), Saifai, Etawah, a tertiary care center serving a predominantly rural catchment area, aimed to define the range of cardiac complications among people with type 2 diabetes attending the outpatient department (OPD). By evaluating prevalence patterns and associating them with modifiable risk factors such as glycosylated hemoglobin (HbA1c) levels, lipid profiles, hypertension, smoking, and lifestyle, the study underscores the need for tailored interventions. Saifai, located in the heart of rural Uttar Pradesh, draws patients from villages spanning Etawah and neighboring districts, providing a representative snapshot of the regional diabetes burden.

The objectives were threefold: (i) to characterize the distribution of cardiac complications; (ii) to analyze associations of cardiac complications with factors known to cause microvascular complications; and (iii) to identify key risk factors through multivariate analysis. By utilizing accessible diagnostic tools such as electrocardiography, treadmill stress testing, and echocardiography, which are applicable even in resource-limited settings, this study aimed to provide valuable insights for primary care physicians.

Understanding these dynamics is crucial not only for clinical management but also for policy formulation. The National Programme for Prevention and Control of Cancer, Diabetes, Cardiovascular Diseases and Stroke (NPCDCS) in India emphasizes screening; however, implementation in rural Uttar Pradesh remains suboptimal [10]. The findings of this study could inform strategies to integrate cardiac risk assessment into routine diabetes clinics, promoting early detection and lifestyle modifications. Moreover, with the rise of telemedicine post-COVID-19, bridging urban-rural divides becomes feasible, potentially revolutionizing care delivery [11].

In summary, this study fills a critical gap in understanding the cardiovascular complications of diabetes mellitus in rural populations, where research remains sparse despite the growing burden of this disease. By elucidating the complex interplay of biological (e.g., hyperglycemia, dyslipidemia), behavioral (e.g., smoking, sedentary lifestyle), and structural (e.g., limited healthcare access) factors, it advocates for a comprehensive strategy to alleviate the cardiovascular toll on underserved communities. The following sections detail the methodology, results, and implications, laying the groundwork for evidence-based interventions to enhance diabetes care in resource-constrained settings.

## Materials and methods

Study setting and oversight

This cross-sectional, hospital-based study was conducted at the Department of General Medicine, UPUMS, Saifai, Etawah, Uttar Pradesh, India, from March 2024 to March 2025. UPUMS in Saifai serves as a referral center for approximately two million rural residents across Etawah and adjacent districts [12], making it an ideal venue for capturing the regional diabetes landscape. The study protocol received approval from the Institutional Review Board of UPUMS (approval number: 2017/105), ensuring adherence to ethical principles outlined in the Declaration of Helsinki [13]. All participants provided written informed consent prior to enrollment, with provisions for illiterate individuals via thumbprint and witness signature. Confidentiality was maintained throughout, with data anonymized and stored securely.

Patient selection

A consecutive sample of 150 patients with type 2 diabetes mellitus (T2DM), diagnosed according to American Diabetes Association (ADA) criteria (fasting plasma glucose ≥126 mg/dL, two-hour postprandial glucose ≥200 mg/dL, or HbA1c ≥6.5%), was recruited from the OPD [14]. Inclusion criteria encompassed adults aged 30-70 years, confirmed T2DM duration of at least one year, and willingness to undergo diagnostic evaluations. Exclusion criteria comprised type 1 diabetes, gestational diabetes, acute infections, pregnancy, end-stage renal disease necessitating dialysis, or any previous history of revascularization procedures. This resulted in a cohort representative of typical OPD attendees, primarily consisting of farmers, laborers, and homemakers from rural areas.

Diagnostic assessments

A comprehensive, multimodal approach was employed to detect cardiac complications, prioritizing non-invasive and cost-effective tools suitable for rural settings.

Clinical evaluation

Detailed medical history and physical examination were performed by a trained physician. History elicited symptoms such as chest pain (angina equivalent), exertional dyspnea, palpitations, orthopnea, or paroxysmal nocturnal dyspnea. Risk factor assessment included duration of diabetes, family history of cardiovascular disease, smoking status (pack-years), alcohol consumption, physical activity levels (sedentary vs. active, defined as <150 minutes/week moderate exercise), and dietary patterns (high-carb, low-vegetable intake typical of rural diets). Physical examination noted blood pressure (using a standardized mercury sphygmomanometer, hypertension defined as ≥140/90 mmHg or on antihypertensive therapy), body mass index (calculated as weight in kg/height in m², obesity ≥25 kg/m² per Asian criteria), and signs of heart failure (jugular venous distension, pedal edema).

Laboratory investigations

Venous blood samples were collected after an overnight fast. HbA1c was quantified using high-performance liquid chromatography on a Bio-Rad D-10 analyzer (Bio-Rad Laboratories, Inc., Hercules, CA, USA), providing a reliable measure of glycemic control over 2-3 months [15]. Lipid profile included total cholesterol, low-density lipoprotein cholesterol, high-density lipoprotein cholesterol, and triglycerides assayed via enzymatic colorimetric methods on a fully automated analyzer (Mindray BS-200; Shenzhen Mindray Bio-Medical Electronics Co., Ltd., Shenzhen, China). Microvascular complications were screened: retinopathy via fundoscopy (simplified grading: none, non-proliferative, proliferative), neuropathy using Michigan Neuropathy Screening Instrument (score ≥2/13 indicative) [16,17], and nephropathy by urine albumin-creatinine ratio (microalbuminuria 30-300 mg/g, macroalbuminuria >300 mg/g) on spot urine samples.

Electrocardiography

A standard 12-lead electrocardiogram (using a GE MAC 5500 machine; GE Healthcare, Milwaukee, WI, USA) was recorded at 25 mm/s speed. Interpretations focused on ischemic changes (ST-T abnormalities, Q waves), arrhythmias (atrial fibrillation, ventricular ectopics), and left ventricular hypertrophy (Sokolow-Lyon criteria).

Treadmill stress test

Patients underwent treadmill stress test per Bruce protocol on a motorized treadmill (GE T2100; GE Healthcare, Milwaukee, WI, USA) under cardiologist supervision [18]. Exercise capacity, heart rate response, and ST-segment depression (>1 mm horizontal/downsloping) were monitored. Positive treadmill stress test indicated inducible ischemia suggestive of CAD.

Echocardiography

Four-dimensional (4D) transthoracic echocardiography (Philips EPIQ 7; Philips Healthcare, Best, The Netherlands) assessed structural and functional parameters. Diastolic dysfunction was diagnosed per American Society of Echocardiography guidelines: E/A ratio (ratio of peak early (E) to late (A) diastolic mitral inflow velocities) <0.8 or >2, deceleration time >240 ms, or E/e' ratio (ratio of peak early mitral inflow velocity (E) to early diastolic mitral annular tissue velocity (e')) >14 [19]. Diabetic cardiomyopathy required a left ventricular ejection fraction <50% without CAD or hypertension as the primary cause. CAD was confirmed by wall motion abnormalities correlating with treadmill stress test/electrocardiogram findings. 

Classification of cardiac complications

Patients were categorized into four mutually exclusive groups: (i) no cardiac complications: normal electrocardiogram, negative treadmill stress test, normal echocardiography; (ii) diastolic dysfunction: echocardiography evidence without systolic impairment or ischemia; (iii) CAD: positive treadmill stress test/electrocardiogram/echocardiography for ischemia; and (iv) diabetic cardiomyopathy: systolic dysfunction independent of CAD/hypertension.

Statistical analysis

Data management and analysis utilized IBM SPSS Statistics version 26 (IBM Corp., Armonk, NY, USA). Continuous variables were expressed as mean ± standard deviation and categorical variables as frequencies and percentages. Baseline characteristics were summarized descriptively. Group comparisons for HbA1c and diabetes duration employed one-way analysis of variance, with Tukey honestly significant difference (HSD) post hoc tests for pairwise differences. Multinomial logistic regression modeled the impact of independent variables (age, body mass index, HbA1c, diabetes duration, lipid parameters, hypertension, microvascular complications, smoking, lifestyle, diet) on complication categories using no cardiac complications as reference. Model goodness-of-fit was assessed via pseudo R-squared and likelihood ratio tests. Variance inflation factors <5 confirmed the absence of multicollinearity. Significance was set at p<0.05. Missing data (one case for retinopathy) underwent listwise deletion, analyzing 149 cases.

This rigorous methodology ensured robust, reproducible findings, with sample size powered to detect 20% prevalence differences (alpha=0.05, power=80%).

## Results

The study enrolled 150 patients, with a mean age of 52.04 ± 10.54 years, reflecting a middle-aged rural cohort vulnerable to chronic diseases. The majority, 93 (62%), were male, consistent with higher OPD attendance among working male patients in patriarchal rural societies. Mean diabetes duration was 4.38 ± 2.89 years, and mean HbA1c was 8.73% ± 2.46%, indicating suboptimal glycemic control overall. Hypertension affected 76 (50.67%)of participants, underscoring its synergy with diabetes in accelerating cardiovascular disease (Table 1).

**Table 1:** Baseline characteristics of study participants. HbA1c: glycosylated hemoglobin; BMI: body mass index; LDL: low-density lipoprotein; HDL: high-density lipoprotein

Characteristic	Value (n=150)
Age (years), mean ± SD	52.04 ± 10.54
Male sex, n (%)	93 (62%)
Female sex, n (%)	57 (38%)
Diabetes duration (years), mean ± SD	4.38 ± 2.89
HbA1c (%), mean ± SD	8.73 ± 2.46
BMI (kg/m²), mean ± SD	24.5 ± 3.2
Hypertension, n (%)	76 (50.67%)
Retinopathy, n (%)	55 (36.91%)
Neuropathy, n (%)	62 (41.33%)
Nephropathy, n (%)	35 (23.33%)
Smoking, n (%)	45 (30%)
Sedentary lifestyle, n (%)	88 (58.67%)
Total cholesterol (mg/dL), mean ± SD	198 ± 42
LDL cholesterol (mg/dL), mean ± SD	128 ± 35
HDL cholesterol (mg/dL), mean ± SD	38 ± 8
Triglycerides (mg/dL), mean ± SD	162 ± 52

**Figure 1:**
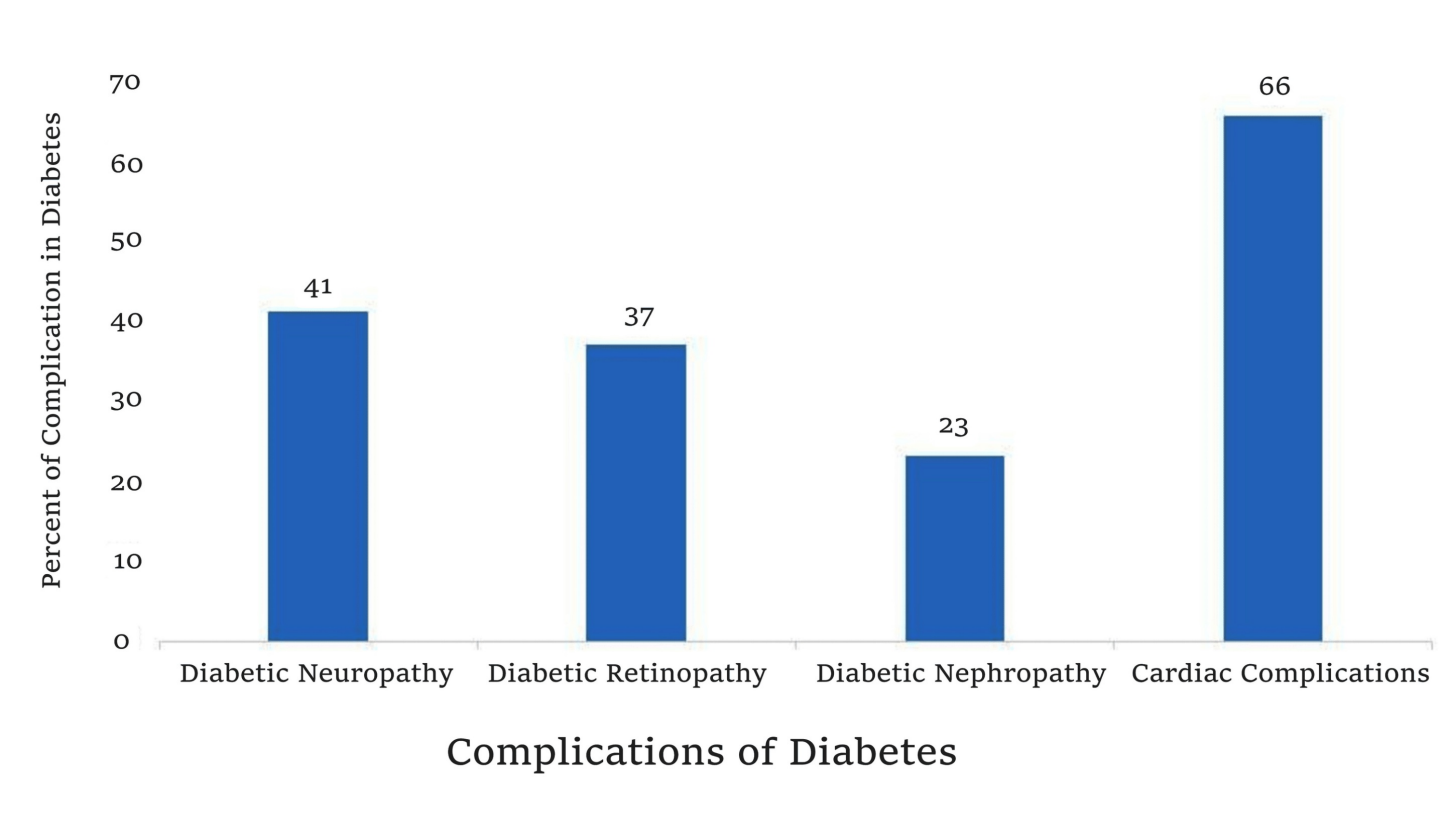
Bar graph showing prevalence of diabetic complications in rural Uttar Pradesh. Cardiac complications (66%) exceeded neuropathy (41%), retinopathy (37%), and nephropathy (23%).

Microvascular complications were common: retinopathy in 55 (36.91%), neuropathy in 62 (41.33%), and nephropathy in 35 (23.33%), highlighting the systemic nature of diabetic vasculopathy (Figure 1).

**Figure 2:**
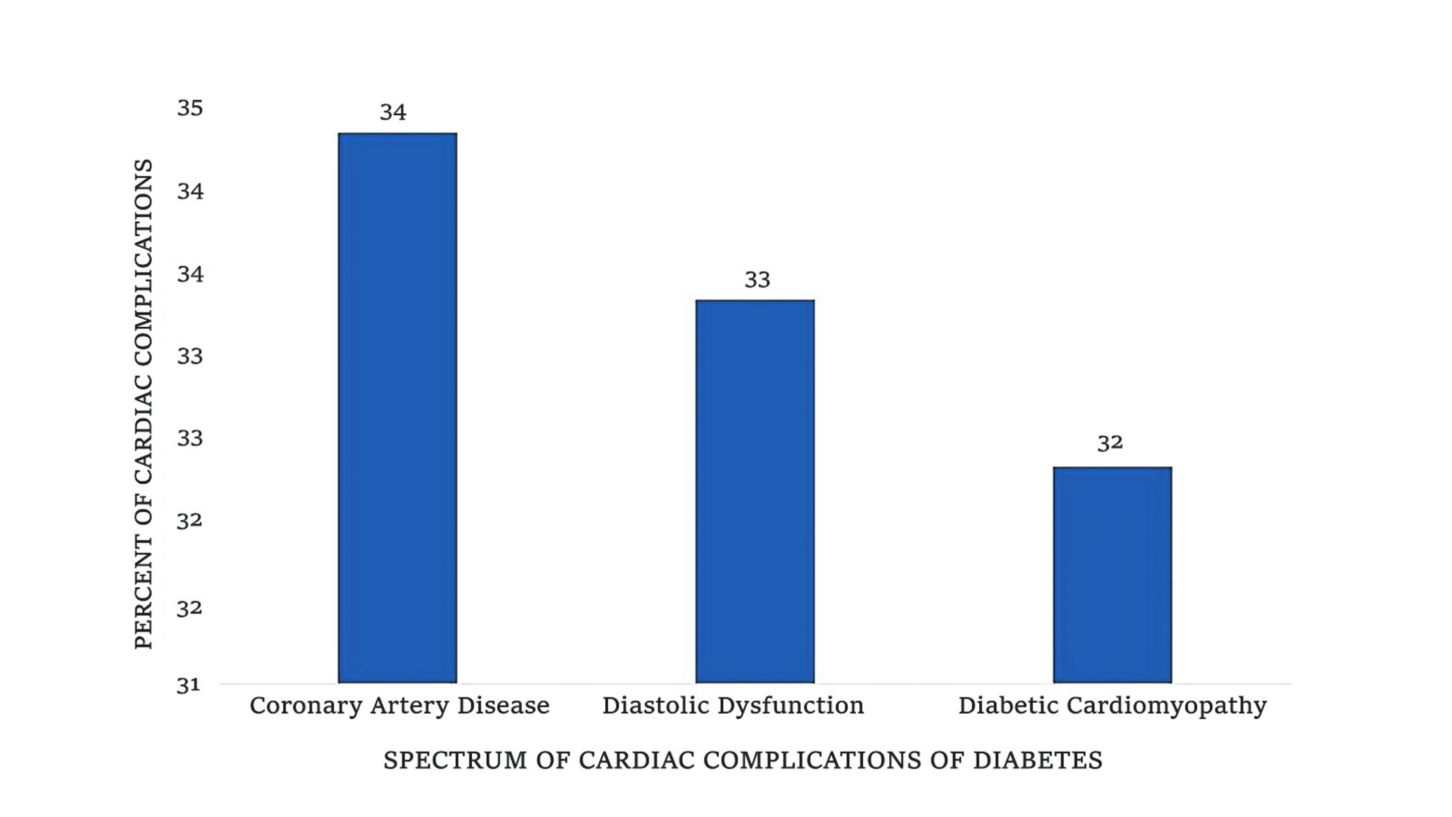
Bar graph showing distribution of cardiac complications among 99 patients with diabetic cardiac disease in rural Uttar Pradesh: coronary artery disease (34%), diastolic dysfunction (33%), and diabetic cardiomyopathy (32%).

Cardiac complications were evident in 99 (66%) of the cohort, highlighting the significant burden on rural diabetic patients, with CAD affecting 34 (34%) patients, diastolic dysfunction impacting 33 (33%) patients, and diabetic cardiomyopathy present in 32 (32%) patients. This distribution reveals CAD as the leading macrovascular issue, closely followed by structural myocardial changes, as illustrated in Figure 2.

**Figure 3:**
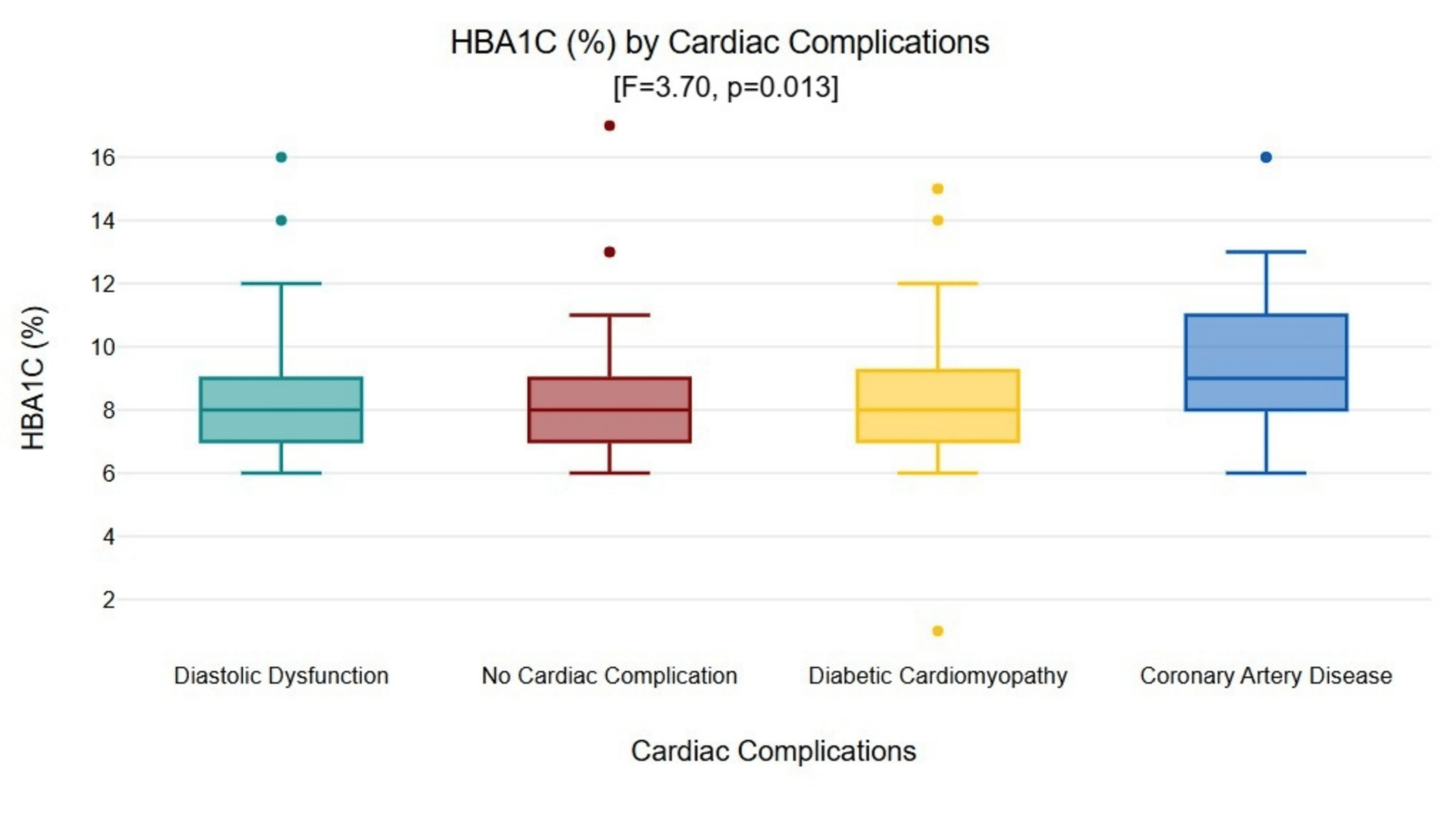
Box plot of HbA1c levels by cardiac complication groups (F=3.70, p=0.013). Coronary artery disease showed significantly higher values vs. no complications (p=0.011). HbA1c: glycosylated hemoglobin

HbA1c varied significantly across complication groups according to analysis of variance (F=3.70, p=0.013), with mean values of 8.31 ± 1.92% for those with no cardiac complications, 8.66 ± 2.73% for diabetic cardiomyopathy, 8.45 ± 2.23% for diastolic dysfunction, and 9.91 ± 2.29% for CAD (Figure 3).

Tukey HSD post hoc tests showed that CAD patients had significantly higher HbA1c compared to those with no cardiac complications (mean difference = 1.598, p=0.011). No significant differences were found among other pairwise comparisons (p>0.05).

**Figure 4:**
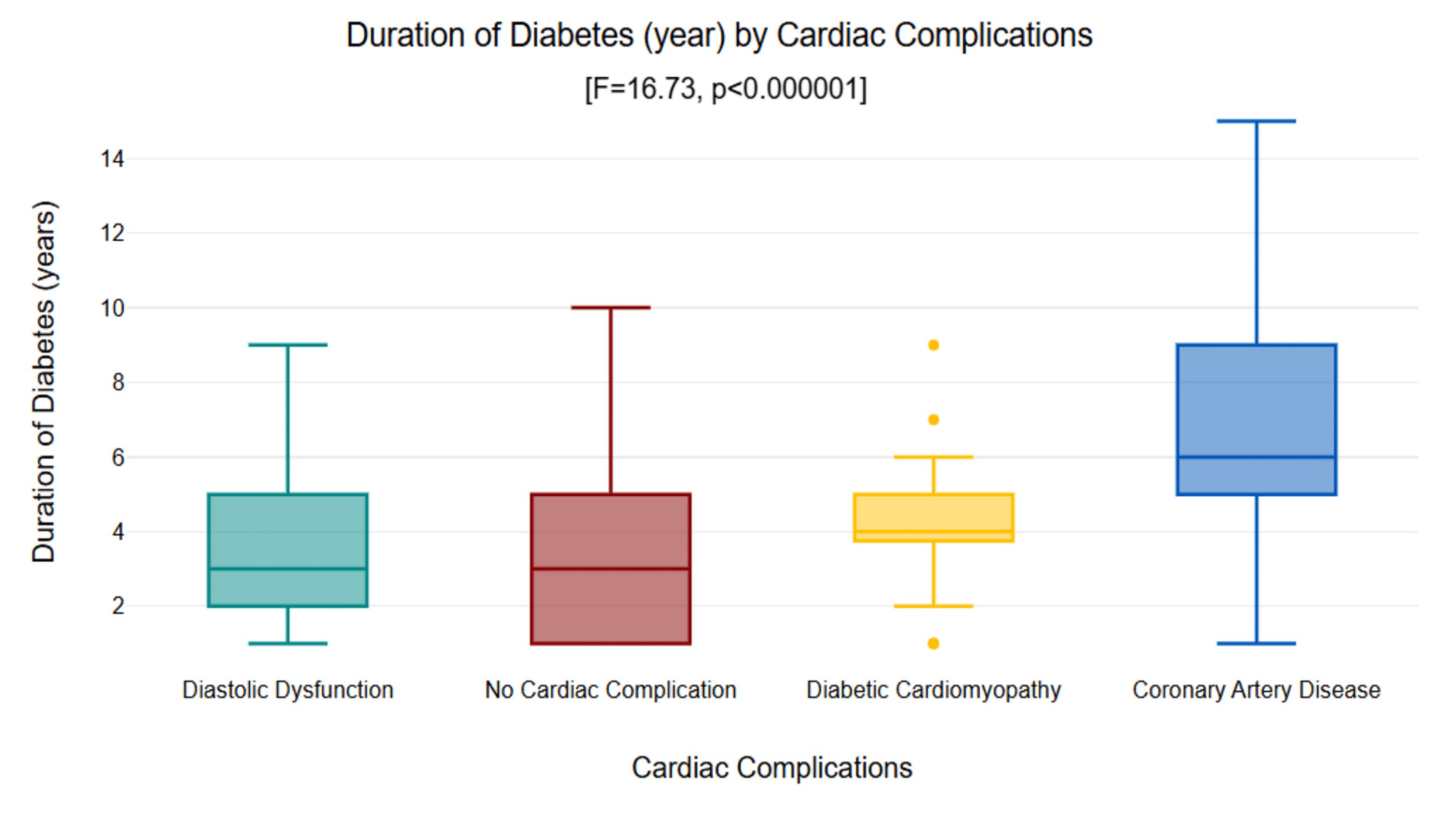
Box plot of diabetes duration by cardiac complication groups (F=16.73, p<0.000001). Coronary artery disease showed significantly longer duration vs. all other groups (p<0.001).

Diabetes duration showed pronounced differences across groups according to analysis of variance (F=16.73, p<0.000001), with means of 3.35 ± 2.30 years for those with no complications, 4.16 ± 1.80 years for diabetic cardiomyopathy, 3.42 ± 1.94 years for diastolic dysfunction, and 7.00 ± 3.66 years for CAD. CAD patients had significantly longer durations of diabetes compared to all other groups (p<0.000001 each), indicating cumulative hyperglycemic exposure as a key factor, as depicted in Figure 4.

The regression model, with a pseudo R² of 0.28 and a p-value of less than 0.000001, identified several predictors for each complication, focusing on modifiable elements. For CAD compared to no complications, significant positive associations included higher HbA1c, low-density lipoprotein cholesterol, triglycerides, total cholesterol, age, diabetes duration, hypertension, smoking, and sedentary lifestyle, while protective effects were noted with higher high-density lipoprotein cholesterol. These findings align with classical cardiovascular disease risk paradigms, where dyslipidemia and behavioral factors amplify ischemic risk in diabetic patients. For diabetic cardiomyopathy compared to no complications, predictors encompassed elevated low-density lipoprotein and total cholesterol, microvascular complications such as retinopathy, neuropathy, and nephropathy, age, diabetes duration, hypertension, and sedentary lifestyle, with high-density lipoprotein cholesterol showing an inverse association. The strong microvascular link suggests shared pathogenic pathways involving endothelial and pericyte damage. For diastolic dysfunction compared to no complications, associations were observed with higher total cholesterol and diabetes duration, alongside lower high-density lipoprotein cholesterol, pointing to early lipid-mediated stiffness in ventricular relaxation. Non-significant factors across categories included body mass index and dietary habits, possibly due to uniform rural dietary patterns characterized by high refined carbohydrates and low fiber. These results illuminate targeted intervention points, particularly for CAD prevention in high-risk rural subgroups.

## Discussion

The results of this study highlight the substantial cardiovascular burden associated with type 2 diabetes in rural Uttar Pradesh, where 99 (66%) patients displayed cardiac complications, a statistic that aligns with global estimates yet surpasses those observed in urban Indian regions [2,20]. The near-equal distribution among CAD of 34 (34%), diastolic dysfunction 33 (33%), and diabetic cardiomyopathy 32 (32%) underscores a diverse spectrum, challenging the notion of a uniform diabetic heart disease phenotype. This heterogeneity likely stems from the interplay of prolonged hyperglycemia, dyslipidemia, and lifestyle adversities inherent to rural existence. 

Central to our observations is the role of glycemic control, as evidenced by elevated HbA1c in CAD patients. Chronic hyperglycemia fosters a pro-thrombotic, pro-inflammatory milieu, accelerating atherosclerosis via advanced glycation end-product formation and nuclear factor-kappa B activation [21]. The UK Prospective Diabetes Study indicated that a 1% elevation in HbA1c was linked to a 14% greater risk of cardiovascular disease [22], supporting the results of our post hoc analysis. In rural Uttar Pradesh, where insulin access is erratic and oral agents like metformin are often self-prescribed, achieving HbA1c <7% remains elusive. Community health workers could bridge this gap through point-of-care HbA1c testing, a low-cost intervention with high yield. 

Diabetes duration proved to be a more robust distinguishing factor, with patients diagnosed with CAD averaging seven years, three times longer than those with uncomplicated cases. This time-related progression indicates gradual myocardial remodeling, where initial diastolic dysfunction develops into systolic failure or ischemia over time [23]. Rural delays in diagnosis, often due to symptom dismissal as gas or stress, perpetuate this trajectory. Comparative studies from southern India report shorter durations for complications (4-5 years), attributable to better urban-rural gradients there [2,24]. Our cohort's extended timelines may highlight Uttar Pradesh's infrastructural deficits. 

The significant role of dyslipidemia in regression analyses warrants further discussion. Elevated levels of low-density lipoprotein, triglycerides, and total cholesterol, combined with reduced high-density lipoprotein, contributed to the risks of CAD and cardiomyopathy, aligning with the findings of the Framingham Heart Study [25]. In diabetic patients, lipoprotein lipase impairment from insulin resistance yields atherogenic remnants, particularly burdensome in high-carb rural diets (rice/potato-heavy, fiber-poor) [26]. The negative high-density lipoprotein association underscores its scavenging role against oxidative low-density lipoprotein. Interventions like statin initiation at primary health centers, per American College of Cardiology/American Heart Association guidelines adapted for low-resource settings, could avert 20-30% of events [27]. Affordability remains a barrier in rural healthcare, but subsidized generics via Jan Aushadhi Kendras offer substantial cost savings (up to 90%) and high customer satisfaction, though low awareness hinders adherence [28]. 

Hypertension, observed in half of our study population, acted synergistically with diabetes to increase CAD odds by threefold, a multiplicative risk consistent with findings from the INTERHEART study - a global case-control study investigating risk factors for acute myocardial infarction [29]. Rural salt intake (10-15 g/day from preserved foods) [30] and stress from agrarian uncertainties likely fuel this. Telemedicine integration with NPCDCS could overcome rural access barriers for blood pressure monitoring [11]. Smoking (30% prevalence) and sedentary lifestyles (59%) further stratified risks, with odds ratios exceeding threefold. A bidirectional causality appears to prevail: diabetes prompts nicotine use for appetite suppression, while inactivity stems from neuropathy-induced mobility limits. Tobacco cessation clinics, leveraging nicotine patches, report 50-60% quit rates; thus, scaling via village health committees is imperative [31]. 

The association of microvascular complications with cardiomyopathy, with odds ratios of approximately three for each, indicates a unified endothelial pathology. Retinopathy signals retinal ischemia mirroring CAD, while nephropathy imposes uremic cardiomyopathy [32]. This clustering advocates holistic screening: a single OPD visit assessing eyes, feet, urine, and heart would help maximize efficiency. 

Contextualizing to rural Uttar Pradesh, our CAD prevalence surpassed urban cohorts (15-20%) in the Indian Council of Medical Research data, likely from advanced presentation [8,26]. Socioeconomic factors, such as a literacy rate of 67% [4] and limited female representation (38% in our sample) due to mobility restrictions, may have influenced the study outcomes. Women, frequently the last to seek medical care, endure the effects of silent ischemia [33]. Implementation of targeted preventive measures in rural areas like Saifai could potentially reduce cardiovascular disease morbidity by 30-40%, restoring vitality to agrarian lives [29].

Strengths of the study include multidiagnostic rigor and multivariate adjustment, minimizing confounders. Limitations include the cross-sectional design, which precludes establishing causality; self-reported lifestyle data that may harbor bias; echocardiography, which is operator-dependent, though standardized; the relatively small sample size (n=150), which may limit statistical power and generalizability; and the single-center, hospital-based nature of the study, which does not incorporate multicentric data and thus may not fully represent diverse rural populations across Uttar Pradesh or India. Data collection in rural settings posed logistical challenges, further constraining the sample size. Future longitudinal studies could assess the impacts of interventions. 

The implications span clinical, policy, and research domains. Clinically, treadmill stress testing and echocardiography should be incorporated into diabetes protocols, targeting an HbA1c <7% and low-density lipoprotein <100 mg/dL. From a policy perspective, strengthening the NPCDCS with rural cardiologists and tele-intensive care unit links is essential [11]. Research avenues should explore randomized trials of yoga for reversing sedentary behavior or fortified staples for managing dyslipidemia. 

Ultimately, this study galvanizes action against the cardiac complications of diabetes in rural India. By demystifying risks, it empowers stakeholders to forge resilient health systems, averting a foreseeable cardiovascular disease epidemic.

## Conclusions

This investigation reveals a stark prevalence of cardiac complications among people with diabetes in the rural areas of Uttar Pradesh, with CAD emerging as a paramount concern fueled by suboptimal glycemic control, extended disease duration, dyslipidemia, hypertension, smoking, and inactivity. Diabetic cardiomyopathy intertwines with microvascular sequelae, while diastolic dysfunction heralds incipient failure. These insights compel urgent paradigms: fortify early screening via accessible diagnostics, enforce aggressive risk modulation, and dismantle access barriers through community-centric models. In rural enclaves like Saifai, such measures could substantially reduce cardiovascular disease morbidity, restoring vitality to agrarian lives. Targeted endeavors in glycemic stewardship, lipid optimization, and lifestyle metamorphosis are non-negotiable for equitable cardiac care in India's diabetes epicenter.
